# Mechanical Thrombectomy for Acute Ischemic Stroke in Octogenarians: A Systematic Review and Meta-Analysis

**DOI:** 10.3389/fneur.2019.01355

**Published:** 2020-01-24

**Authors:** Weisong Zhao, Pengju Ma, Ping Zhang, Xuejing Yue

**Affiliations:** ^1^Department of Pediatrics, The First Clinic College of Xinxiang Medical University, Xinxiang, China; ^2^Department of Neurosurgery, The First Affiliated Hospital of Xinxiang Medical University, Xinxiang, China; ^3^Department of Neurology, The First Affiliated Hospital of Xinxiang Medical University, Xinxiang, China; ^4^School of Basic Medicine, Xinxiang Medical University, Xinxiang, China

**Keywords:** stroke, elderly, mechanical thrombectomy, outcomes, meta-analysis

## Abstract

**Background and purpose:** Multiple randomized trials have confirmed that mechanical thrombectomy (MT) is an effective treatment method for patients with acute ischemic stroke (AIS). However, evidence on the safety and efficacy of MT in elderly patients compared with younger patients is controversial. This meta-analysis is aimed to systematically compare the outcomes of elderly patients and younger patients after MT for AIS.

**Methods:** A systematic literature search was conducted through the PubMed, EMBASE, and Cochrane Library database. The primary outcomes were favorable functional outcome at 90 days and mortality. The secondary outcomes were symptomatic intracerebral hemorrhage (sICH) and successful recanalization rate. Odds ratios (ORs) were estimated using a random effects model.

**Results:** Sixteen studies published between 2014 and 2019 were included in this meta-analysis totally involving 3,954 patients. The pooled results showed that patients aged ≥80 years had worse functional outcome (OR = 0.40; 95% CI, 0.32–0.50; *P* < 0.001) and higher rates of mortality (OR = 2.26; 95% CI, 1.73–2.95; *P* < 0.001). There was a trend of higher rates of sICH in patients aged ≥80 years compared with patients aged <80 years, whereas this did not reach statistical significance (OR = 1.28; 95% CI, 0.89–1.84; *P* = 0.18). Furthermore, the frequency of successful recanalization was also lower in patients aged ≥80 years compared with patients aged <80 years (OR = 0.72; 95% CI, 0.55–0.95; *P* = 0.02). The subgroup analysis indicated that in comparison with those studies published between 2014 and 2016, elderly patients undergoing MT had better outcomes in studies published between 2017 and 2019.

**Conclusion:** Elderly patients undergoing MT had higher risk of mortality and worse functional outcome. Meanwhile, there was a trend toward higher rates of sICH and lower probability of achieving successful recanalization in elderly patients. These findings emphasize the need for improving the rates of successful recanalization in elderly patients with AIS. In addition, advanced technology of endovascular intervention and peri-interventional management might be associated with the prognosis in elderly patients. However, more prospective or randomized studies should be conducted to further explore this issue.

## Introduction

With the development of healthcare, the percentage of elderly in the total population is growing rapidly. Undoubtedly, the aging of the population will increase the incidence of chronic diseases and impose personal and society's medical burden. Currently, stroke has become the second leading cause of death next to heart diseases, which killed 5.5 million persons per year (1). According to statistics, the aging of population was one of the major causes of increased incidence of stroke (2).

Over the past decade, the management regarding acute ischemic stroke (AIS) has changed significantly. In 2013, three randomized trials reported that in comparison with standard medical treatments, mechanical thrombectomy (MT) using the first-generation devices for patients with AIS failed to provide better outcomes (3–5). However, in 2015, five randomized trials reported a clear benefit of MT using the second-generation devices for patients with AIS than standard medical treatments (6–10). Currently, MT has been widely accepted as the first-line therapy for patients with proximal and relevant emergent large vessel occlusion. The HERMES meta-analysis that pooled the individual patient data from the above five trials has shown a consistent benefit of MT in other potential patient populations, even in those patients aged ≥80 years (11).

With regard to the safety and efficacy of MT in the elderly population, there is a lack of high-quality evidence from large randomized trials, and the results from observational studies are discrepant. Recently, Sharobeam et al. performed a cohort study based on a prospective database and a meta-analysis that included 14 studies (12). Their study indicated that elderly patients undergoing MT had worse functional outcomes and higher likelihood of mortality than younger patients (12). However, their study failed to assess the difference of other outcomes such as symptomatic intracerebral hemorrhage (sICH), successful recanalization rate, onset to groin puncture time, and groin puncture to recanalization time between the elderly patients and younger patients (12). Therefore, we sought to perform a formal meta-analysis of available data to systematically compare the safety, efficacy, and outcomes of MT in the elderly population (aged ≥80 years) with younger population (aged <80 years).

## Materials and Methods

This meta-analysis was conducted according to the recommendations of the preferred reporting items for systematic reviews and meta-analyses (PRISMA) guidelines (13).

### Literature Search

A comprehensive literature search was conducted through PubMed, EMBASE, and the Cochrane Library database to October 9 2019, using the following keywords: ischemic stroke; cerebral infarct; elderly; octogenarians; 80 or older; mechanical thrombectomy; thrombectomy; endovascular therapy; outcomes; hemorrhage; death; and mortality. Furthermore, we hand-searched the references of these eligible articles to identify other potentially relevant studies. This process was conducted by two authors (Weisong Zhao and Pengju Ma) independently, and any difference was addressed through a full discussion with a third author (Xuejing Yue).

### Outcomes and Study Selection

The primary end points of this study were favorable functional outcome at 90 days [defined as a modified Rankin scale (mRS) score of 0–2] and mortality. The secondary outcomes were sICH and successful recanalization rate. Two authors (Weisong Zhao and Pengju Ma) independently screened all titles, abstracts, and subsequently read the full text for possible eligibility. If there was any discrepancy, a consensus was reached through consulting a third author (Ping Zhang). Study included in this meta-analysis must meet the following criteria: (a) patients were diagnosed with AIS; (b) the second-generation thrombectomy device and/or aspiration catheter was used for MT; (c) studies reported information on functional outcome at 90 days and mortality; (d) the elderly group was defined as patients aged ≥80 years, and younger group was defined as patients aged <80 years. Studies were excluded if they met the following criteria: (a) non-English language; (b) duplicated articles, review articles, or case reports; (c) single-arm study; (d) outdated thrombectomy device (primarily Merci device) was used for MT; (e) partial patients undergoing thrombolytic therapy only.

### Data Extraction and Quality Assessment

For each eligible study, the following data were extracted by two authors (Weisong Zhao and Pengju Ma): first author, number of patients, year of publication, study type, devices used for MT, number of females, mean age, mean admission NIHSS, groin puncture to revascularization time (GTR), onset to groin puncture time (OTP), favorable functional outcome at 90 days, mortality, sICH, and successful recanalization rate. The Newcastle-Ottawa Scale (NOS) was used to assess the quality of each eligible study, with a NOS score ≥7 points, indicating high quality. If there was any discrepancy, a consensus was reached through consulting a third author (Xuejing Yue).

### Statistical Analysis

The statistical analysis was performed by using Review Manager 5.3 software. Odds ratios (ORs) and 95% confidence intervals (CIs) were calculated using a random effects model. The heterogeneity across studies was evaluated by using the I-squared (I^2^) statistics index. *I*^2^ more than 50% indicated significant heterogeneity existing across the eligible studies. In addition, visual funnel plots were used to evaluate the publication bias in this meta-analysis.

## Results

### Literature Search, Study Characteristics, and Quality Assessment

A total of 1,810 relevant studies were initially identified through literature retrieval, and 16 studies were finally included in this meta-analysis after screening (12, 14–28). A flow diagram of the detailed search process was present in Supplemental Figure 1. Among the 16 studies, three were multicenter studies and others were single-center studies. Five studies were published between 2014 and 2016, and 11 studies were published between 2017 and 2019. Data on 3,954 patients were finally pooled, of whom 1,059 patients were aged ≥80 years and 2,895 patients were aged <80 years. The detailed baseline characteristics and outcomes of each study were presented in Tables 1, 2, respectively. Funnel plots indicate no significant publication bias was found among the 16 studies (Supplemental Figures 2–5). In addition, the NOS scores of each study ranged from 6 to 8, suggesting a moderate and high quality of all included studies (Supplemental Table 1).

**Table 1:** Characteristics of studies included in this meta-analysis.

**References**	**Study type**	**Devices used** ** for MT**	**Age** **≥80**					**Age** ** <80**				
			**Median** ** age, years**	**No. of** ** female**	**NIHSS** ** (Mean)**	**GTR** ** minutes**	**OTG,** ** minutes**	**Median** ** age, years**	**No. of** ** female**	**NIHSS** ** (Mean)**	**GTR** ** minutes**	**OTG,** ** minutes**
Castonguay et al. (18)	M, R	Solitaire-FR	85.2	51	18.9	38.2	358.9	62.2	127	17.9	31.4	364.7
Parrilla et al. (25)	S, R	Solitaire-FR	NA	22	9.7	74.5	298	NA	50	6.5	63	280
Broussalis et al. (17)	S, R	Stent-retriever	82.5	13	18	78	180	65.2	59	18	87	195
Kleine et al. (28)	S, R	Stent-retriever	NA	NA	15	58	199	NA	NA	15	66	206
Cohen et al. (19)	S, R	Stent-retriever	84.3	9	18.4	40	239.5	63.1	28	18.2	44.5	232.2
Son et al. (23)	S, R	Stent retriever and FAST	82	21	18	54	234	67.5	74	16	62	278.8
Sallustio et al. (27)	S, R	Stent-retriever and aspiration catheter	84.9	49	18	60	219.0	64.5	76	18	78	239.7
Tajima et al. (24)	S, R	Stent retrievers and Penumbra reperfusion system	84.7	14	22.4	56	251	69.6	12	20.2	65	207
Azkune et al. (16)	S, R	Solitaire FR	85.2	19	16.6	NA	206.3	67.7	14	13.6	NA	209.7
Figueiredo et al. (20)	S, R	Stent-retriever and aspiration catheter	84	22	17	NA	NA	65	56	17	NA	NA
Imahori et al. (21)	S, R	Stent-retriever and aspiration catheter	85	19	15	NA	205	73	18	15	NA	212
Karhi et al. (22)	S, R	Aspiration catheter and stent-retriever	84.0	29	14.1	68	221	63.2	47	12.7	32	306
Koizumi et al. (26)	M, R	Stent-retriever and Penumbra reperfusion system	84	41	19	50	199	70	67	17	50	207
Alawieh et al. (14)	S, R	Aspiration first pass technique and Stent-retriever	84.7	74	17.3	NA	413.9	69.7	103	15.8	NA	459.5
Sharobeam et al. (12)	S, R	Stent-retriever and aspiration catheter	85	39	18	35	270	64	51	17	31	274
Alawieh et al. (15)	M, R	Aspiration catheter and stent-retriever	85	230	17	45	369	62	474	16	48	399

*S, single-center; M, multi-centers; R, retrospective; NA, no report; Y, year; GTR, groin puncture to recanalization time; OTG, onset to groin puncture time; MT, mechanical thrombectomy; NIHSS, National Institutes of Health Stroke Scale*.

**Table 2:** Outcomes of studies included in this meta-analysis.

**References**	**Total** ** patients**	**Age** **≥80**					**Age** ** <80**				
		**No. of** ** patients**	**90 days** ** mRS ≤2**	**No. of** ** sICH**	**No. of** ** mortality**	**Successful** ** recanalization**	**No. of** ** patients**	**90 days** ** mRS ≤2**	**No. of** ** sICH**	**No. of** ** mortality**	**Successful** ** recanalization**
Castonguay et al. (18)	356	78	18	10	29	54	276	113	25	68	201
Parrilla et al. (25)	150	34	4	2	12	30	116	58	3	20	109
Broussalis et al. (17)	166	28	5	5	10	19	138	66	14	16	99
Kleine et al. (28)	125	40	5	1	13	17	85	47	0	10	70
Cohen et al. (19)	71	16	3	2	6	14	55	27	1	4	47
Azkune et al. (16)	81	31	16	5	6	29	50	32	2	5	48
Son et al. (23)	207	34	15	2	1	28	173	108	6	20	148
Sallustio et al. (27)	219	62	19	7	25	NA	157	54	22	46	NA
Tajima et al. (24)	78	25	11	2	2	24	53	34	5	6	47
Figueiredo et al. (20)	141	35	21	0	5	31	106	69	3	9	99
Imahori et al. (21)	80	36	15	1	3	30	44	25	2	2	41
Karhi et al. (22)	199	37	10	NA	17	28	162	84	NA	16	121
Koizumi et al. (26)	221	78	27	3	6	62	143	73	8	11	112
Alawieh et al. (14)	335	108	22	6	37	97	227	100	14	45	212
Sharobeam et al. (12)	181	71	20	4	19	68	110	61	5	18	107
Alawieh et al. (15)	1,346	346	65	NA	118	130	1,000	398	NA	184	404

*mRS, modified Rankin Scale; sICH, symptomatic intracerebral hemorrhage*.

### Functional Outcome at 90 Days

**Figure 1:**
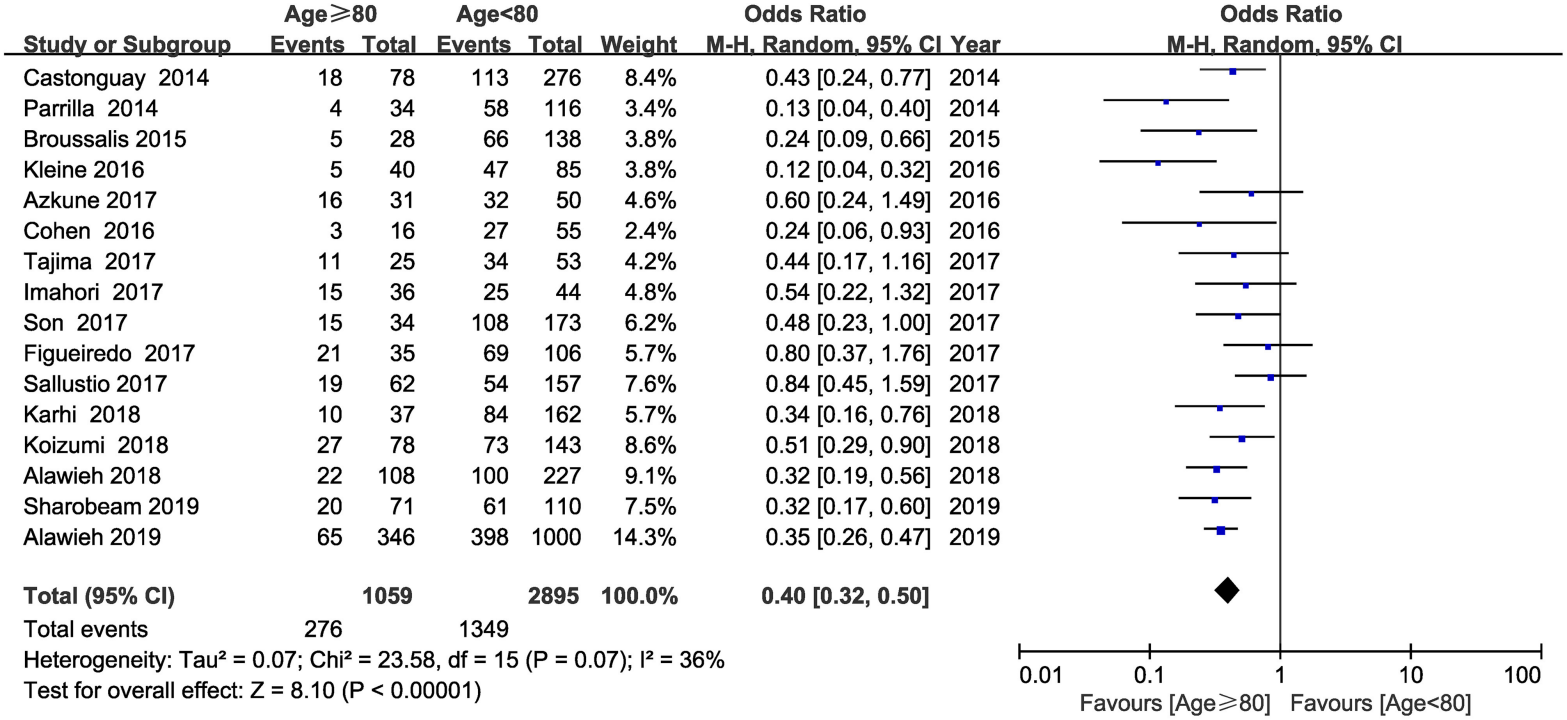
Forest plot of favorable functional outcome at 90 days.

Information on functional outcome at 90 days was available from the 16 studies. The pooled results showed that elderly patients undergoing MT had worse functional outcome than younger patients (OR = 0.40; 95% CI, 0.32–0.50; *P* < 0.001) (Figure 1). No substantial heterogeneity was detected between the 16 studies (*I*^2^ = 36, *P* = 0.07).

### Mortality

**Figure 2:**
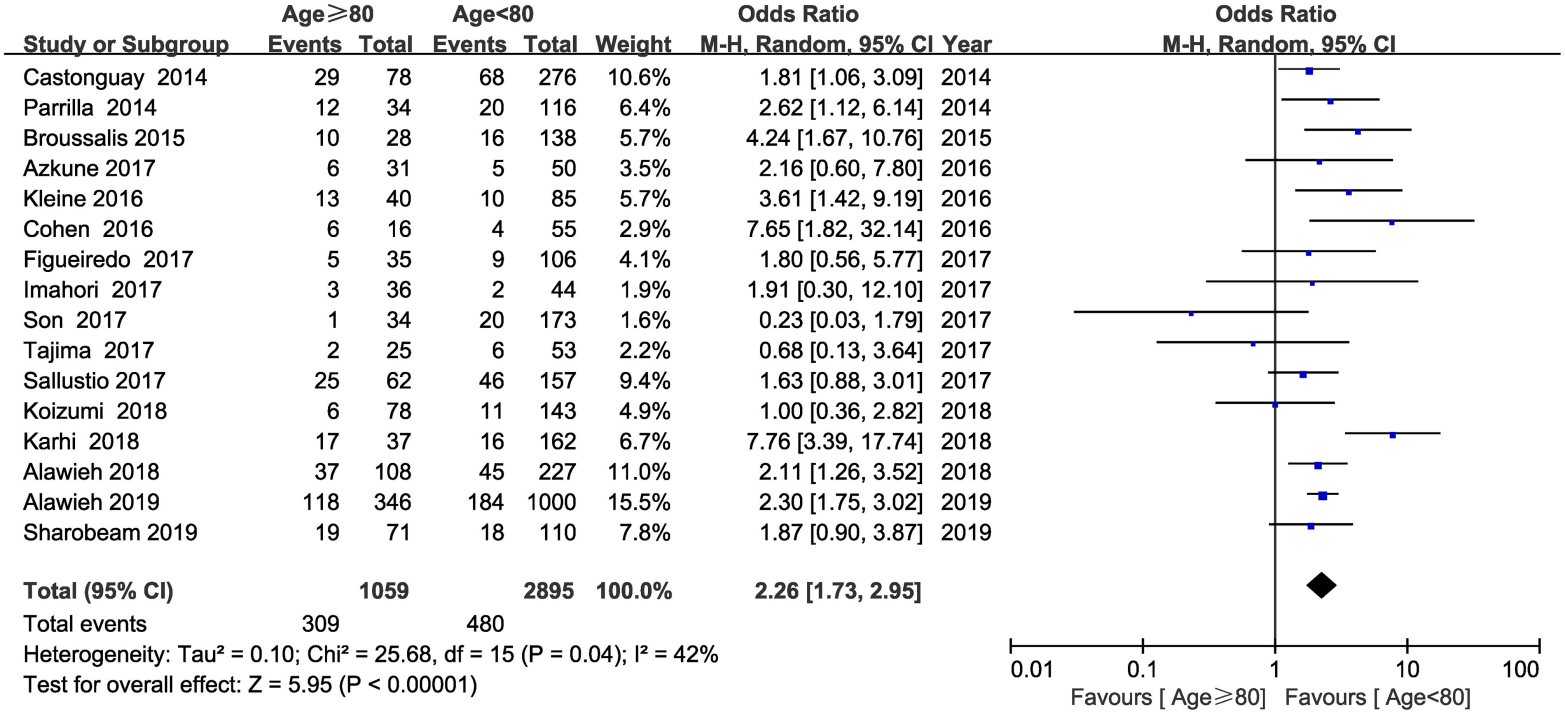
Forest plot of mortality.

Sixteen studies reported mortality within 90 days and were included in this analysis. The pooled results showed that elderly patients treated with MT had a higher likelihood of mortality than younger patients (OR = 2.26; 95% CI, 1.73–2.95; *P* < 0.001) (Figure 2). No substantial heterogeneity was detected across the 16 studies (*I*^2^ = 42, *P* = 0.04).

### Symptomatic Intracerebral Hemorrhage (sICH)

**Figure 3:**
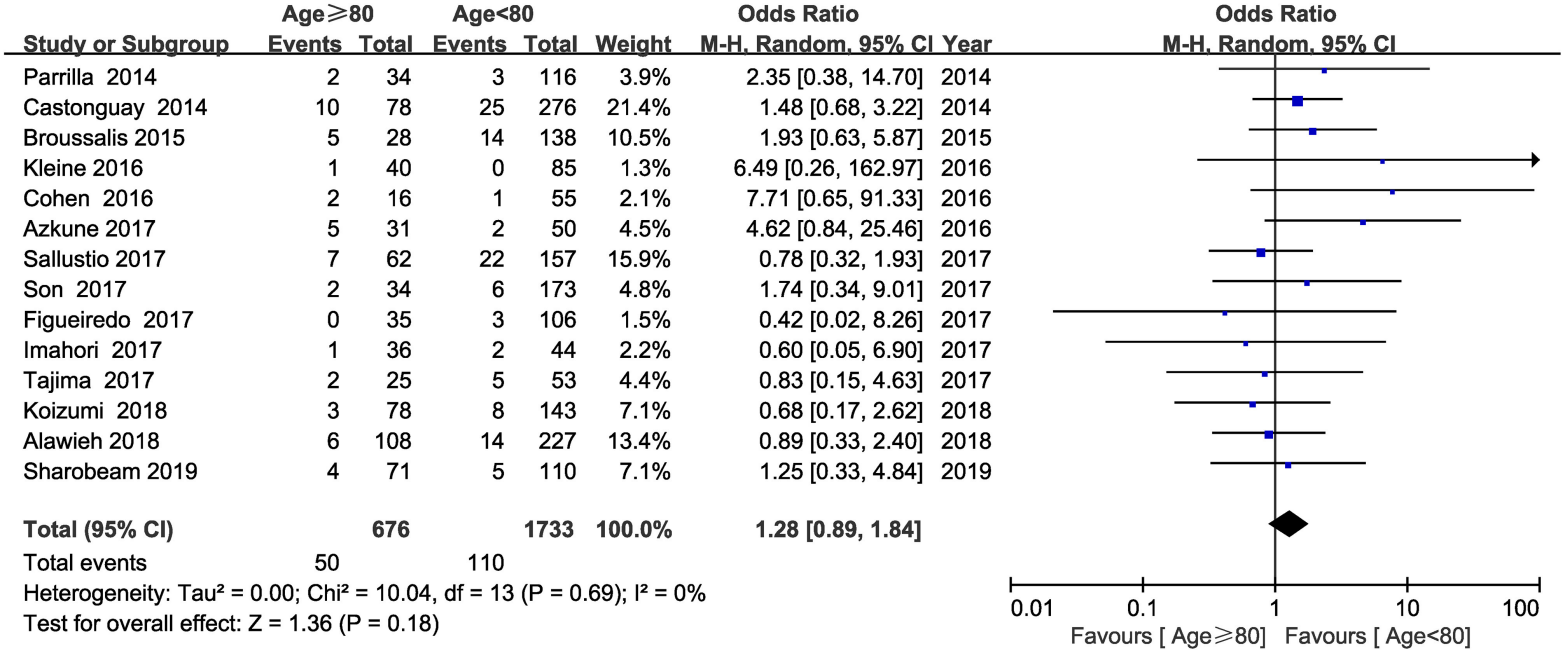
Forest plot of symptomatic intracerebral hemorrhage (sICH).

Fourteen studies involving 2,409 patients reported the information regarding sICH. The pooled results indicated that sICH was seen more frequently in patients aged ≥80 years; however, this did not reach statistical significance (OR = 1.28; 95% CI, 0.89–1.84; *P* = 0.18) (Figure 3). No heterogeneity was detected across the fourteen studies (*I*^2^ = 0, *P* = 0.69).

### Successful Recanalization Rate

**Figure 4:**
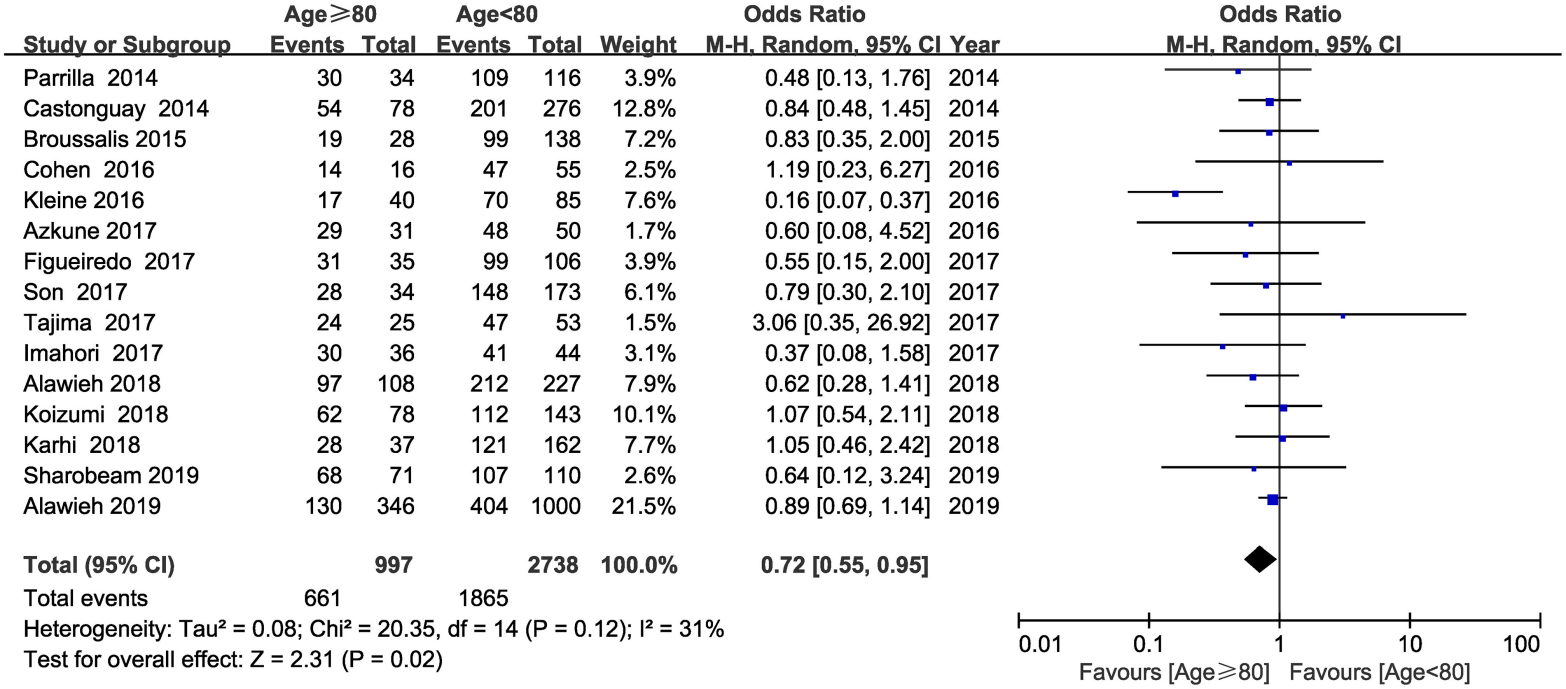
Forest plot of successful recanalization rate.

Data on 3,735 patients from 15 studies were used in the analysis of successful recanalization rate. The pooled results indicated that successful recanalization rate was lower in patients aged ≥80 years compared with patients aged <80 years (OR = 0.72; 95% CI, 0.55–0.95; *P* = 0.02) (Figure 4). No substantial heterogeneity was detected across the 16 studies (*I*^2^ = 31, *P* = 0.12).

## Discussion

Patients aged ≥80 years have usually been excluded from previous randomized trials because of their uncertain risk. Therefore, only 198 patients aged ≥80 years were included in the five famous randomized trials (6–10). In the HERMES meta-analysis, the rates of functional outcomes at 90 days and mortality for those patients undergoing MT were 46 and 15%, respectively (11). Among those patients aged ≥80 years, the rates of functional outcomes at 90 days and mortality were 29.8 and 28%, respectively (11). Among those patients aged ≥80 years but not undergoing MT, the rates of functional outcomes at 90 days and mortality were 13.9 and 45%, respectively (11). In 2018, Hilditch et al. conducted a meta-analysis and pooled the incidence of several major outcomes in 860 elderly patients undergoing MT and compared them with the results in elderly patients without MT from HERMES meta-analysis (29). Their pooled results showed that elderly patients had higher rates of achieving favorable functional outcome and lower rates of mortality than those patients without MT, even with more incidence of sICH and complications (29). The above evidence showed that elderly patients treated with MT had worse outcomes than younger patients treated with MT but still better than those patients who were not treated with MT (11, 12).

Although age has been demonstrated as a negative independent factor for the outcomes of AIS in many studies, other features such as sICH, successful recanalization rate, initial NIHSS score, and time of operation were also associated with the prognosis of AIS. Our study including 16 observational studies totally involving 3,954 patients aimed to systematically evaluate the differences in outcomes between the elderly patients and younger patients after MT for AIS. The pooled results showed that elderly patients undergoing MT were associated with higher risk of mortality and worse functional outcome. The rates of sICH in patients aged ≥80 years was found higher than those in patients aged <80 years but did not reach statistical significance. In addition, the rates of successful recanalization were found lower in patients aged ≥80 years compared with those in patients aged <80. Potential explanations for the worse functional outcome and higher rates of mortality in elderly patients treated with MT are as follows: first, the age itself was a negative independent factor for prognosis; the neurological reserve and neuroplasticity in brain tissues were decreased with age, which could delay the recovery of patients (11, 30, 31); Second, elderly patients are usually accompanied by higher rates of in-hospital complications, such as intracerebral hemorrhage and other diseases in old age (14, 16, 19, 32). Finally, successful recanalization was an influential factor for achieving favorable function outcome, particularly in elderly patients (21). In this meta-analysis, elderly patients had lower rates of successful recanalization in comparison with younger patients; one possible reason is that elderly patients usually have increased vessel tortuosity, which may decrease the effectiveness of thrombectomy device. The trend toward higher rates of sICH in elderly patients can be caused by a high incidence of vascular sclerosis, hypertension, and other vascular diseases in elderly patients. Furthermore, elderly patients often have a higher prevalence of leukoaraiosis, which may be associated with the increased risk of sICH (33, 34).

According to the pooled data of our meta-analysis, no significant difference was found in mean baseline National Institutes of Health Stroke Scale (NIHSS) score (17.0 vs. 15.9), onset to groin puncture time (257.6 vs. 271.3), and groin puncture to recanalization time (54.7 vs. 54.8) between elderly patients and younger patients. In addition, we conducted a subgroup analysis according to the publication years (Supplemental Table 2). The results indicated that in comparison with those studies published between 2014 and 2016, elderly patients undergoing MT had lower rates of sICH and mortality and were more prone to achieve favorable functional outcomes at 90 days and successful recanalization in studies published between 2017 and 2019. We presumed that the technology advances in endovascular intervention and more perfect nursing measurement might be the reasons to improve the prognosis in elderly patients. However, we cannot perform further analysis to confirm our presumption because relevant data such as how the peri-interventional management and surgical process took place are not available. Further studies should be conducted to explore this issue and pay attention to what kind of postoperative management is better for the elderly population. In addition, several limitations of this study should be noted. First, all studies included in this meta-analysis were retrospective, which could introduce the risk of selection bias. Second, there were also variations on the mode of anesthesia, concomitant use of thrombolytic therapy, stroke subtype, and etiology of stroke across the 16 eligible studies, all of which could affect the outcomes of patients undergoing MT. Owing to the lack of data from some studies, it was impossible to perform further analysis according to these inconsistent data. Third, patients with lower premorbid mRS are more likely to benefit from MT, whereas this information is not available in most studies and the pooled results of our meta-analysis cannot be adjusted according to it. Finally, publication bias could also affect the clinical outcomes between elderly patients and younger patients because more experienced centers or centers with better results in treating elderly patients with AIS are more likely to report their results. However, funnel plots indicate that no significant publication bias was found among the 16 studies.

## Conclusion

Our meta-analysis is based on 16 retrospective studies totally enrolling 3,954 patients, which indicated that patients aged ≥ 80 years undergoing MT were associated with higher risk of mortality and worse functional outcome. In addition, patients aged ≥ 80 years after MT had lower probability of achieving successful recanalization and a trend of higher rates of sICH than patients aged < 80 years. Additional prospective and randomized studies are necessary to focus on how to improve the rates of successful recanalization in elderly patients with AIS and determine what kind of postoperative management and technology of endovascular intervention are better for elderly patients being considered for MT.

## Data Availability Statement

The datasets generated for this study are available on request to the corresponding author.

## Author Contributions

WZ and XY contributed to literature search, data analysis, and drafting and revision of the manuscript. PM and PZ contributed to data collection and crafting and revision of the tables and figures.

### Conflict of Interest

The authors declare that the research was conducted in the absence of any commercial or financial relationships that could be construed as a potential conflict of interest.
