# Supplementary material for: Mechanical Thrombectomy for Acute Ischemic Stroke in Octogenarians: A Systematic Review and Meta-Analysis
Source: Front Neurol. 2020 Jan 24;10:1355. doi: 10.3389/fneur.2019.01355 (PMC6993560; doi:10.3389/fneur.2019.01355)
Supplement: Supplementary file 1 [file Table_1.DOCX]

Supplementary Material

1 Supplementary Figures and Tables

1.1:

**Supplemental Figure 1:** A flow diagram demonstrating the study selection process.

**Supplemental Figure 2-5:** Funnel plots of included studies.

**
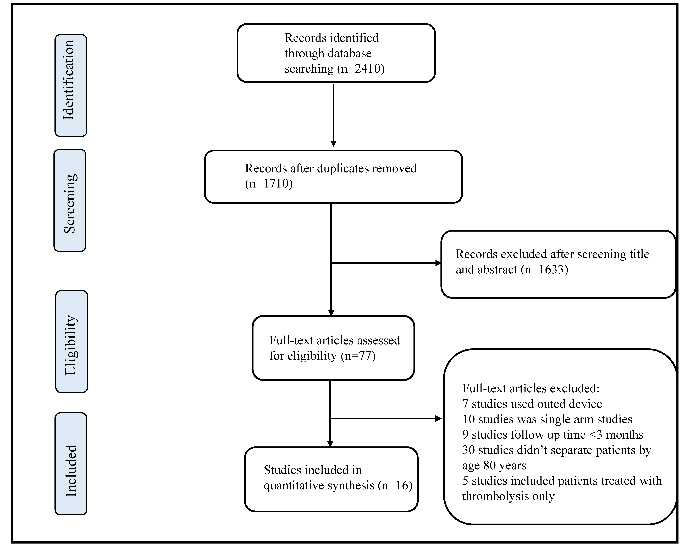
Supplemental Figure 1**


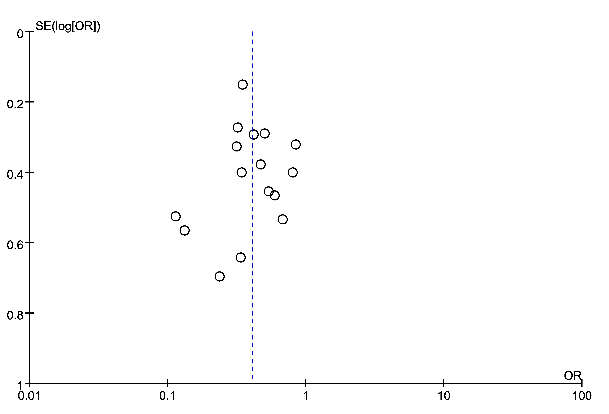
**
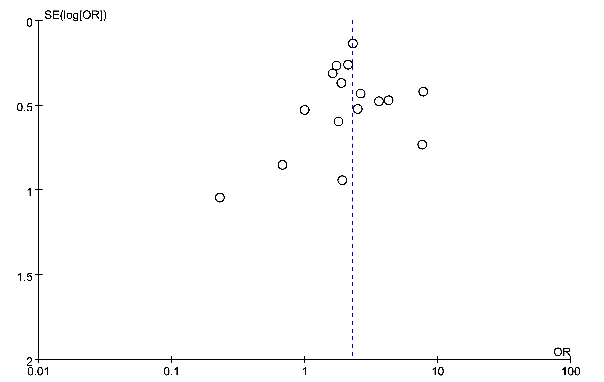
Supplemental Figure 2-5**


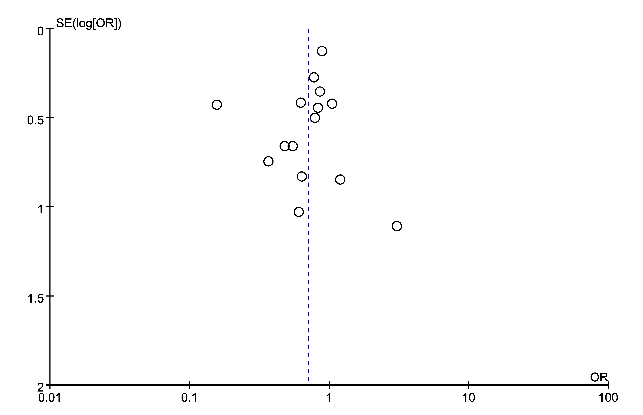


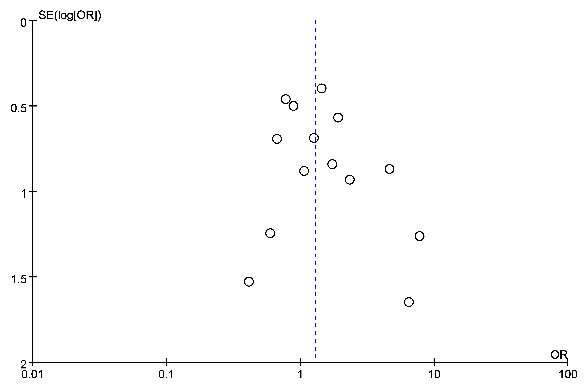


1.2

Supplemental Table 1: Quality assisment of studies included in this Meta-Analysis.

Supplemental Table 2: Subgroup analysis according to the publication years.

**Supplemental Table 1: Quality Assisment of Studies Included for Meta-Analysis.**

| Study | Selection | | | | Comparability | Outcome |  | | Score |
| --- | --- | --- | --- | --- | --- | --- | --- | --- | --- |
|  | Representativeness of the exposed cohort | Selection of the nonexposed cohort | Ascertainment of exposure | Demonstration that outcome of interest was not present at start of study | Comparability of controls on the basis of the design or analysis | Assessment of outcome | Was follow up long enough for outcomes to occur | Adequacy of follow up of cohorts | Total scores |
| Castonguay 2014 | 1 | 0 | 1 | 1 | 1 | 0 | 1 | 1 | 6 |
| Parrilla 2015 | 1 | 1 | 1 | 1 | 1 | 1 | 1 | 1 | 8 |
| Broussalis 2015 | 0 | 0 | 1 | 1 | 1 | 1 | 1 | 1 | 6 |
| Kleine 2016 | 1 | 1 | 0 | 1 | 1 | 1 | 1 | 1 | 7 |
| Cohen 2016 | 1 | 1 | 1 | 1 | 1 | 0 | 1 | 1 | 7 |
| Azkune 2017 | 1 | 0 | 1 | 1 | 1 | 1 | 1 | 1 | 7 |
| Son 2017 | 1 | 1 | 1 | 1 | 1 | 1 | 1 | 1 | 8 |
| Sallustio 2017 | 1 | 1 | 1 | 1 | 1 | 1 | 1 | 1 | 8 |
| Tajima 2017 | 1 | 1 | 1 | 1 | 1 | 1 | 1 | 1 | 8 |
| Figueiredo 2017 | 1 | 1 | 1 | 1 | 1 | 0 | 1 | 1 | 7 |
| Imahori 2017 | 0 | 0 | 1 | 1 | 1 | 1 | 1 | 1 | 6 |
| Karhi 2018 | 1 | 1 | 0 | 1 | 1 | 1 | 1 | 1 | 7 |
| Koizumi 2018 | 1 | 1 | 1 | 0 | 1 | 1 | 1 | 1 | 7 |
| Alawieh 2018 | 1 | 1 | 1 | 1 | 1 | 1 | 1 | 1 | 8 |
| Sharobeam 2019 | 1 | 1 | 0 | 0 | 1 | 1 | 1 | 1 | 6 |
| Alawieh 2019 | 1 | 1 | 1 | 1 | 1 | 1 | 1 | 1 | 8 |

**Supplemental Table 2: Subgroup analysis according to the publication years.**

|  | Functional outcome | I^2^ | Mortality | I^2^ | sICH | I^2^ | Successful recanalization rate | I^2^ |
| --- | --- | --- | --- | --- | --- | --- | --- | --- |
| 2014-2016 | 0.24(0.13-0.42) | 39 | 2.89(1.81-4.62) | 32 | 1.87(1.05-3.34) | 0 | 0.54(0.26-1.10) | 66 |
| 2017-2019 | 0.45(0.36-0.56) | 22 | 2.00(1.43-2.81) | 48 | 1.01(0.64-1.61) | 0 | 0.85(0.69-1.04) | 0 |
